# Feasibility of video assessment of operative skills in FCPS vascular surgery

**DOI:** 10.12669/pjms.39.1.6737

**Published:** 2023

**Authors:** Ahsin Manzoor Bhatti, Sadia Ahsin, Sumreena Mansoor

**Affiliations:** 1 Ahsin Manzoor Bhatti, Department of Vascular Surgery, CMH Rawalpindi, Pakistan; 2 Sadia Ahsin, Department of Physiology, Foundation University, Islamabad, Pakistan; 3 Sumreena Mansoor, Department of Biochemistry, Shifa Tameer-e-Milat University, Islamabad, Pakistan

**Keywords:** Surgical Procedures, Operative / education, Surgical Procedures, Operative / standards Assessment

## Abstract

**Objective:**

To explore the feasibility of assessment of operative skills of FCPS vascular surgery trainees based on video recordings of the surgical procedures with a view to introduce it in the curriculum.

**Methods:**

This qualitative study was carried out from 9^th^ April 2021 – 15^th^ July 2021 at Shifa Tameer e Millat University, Islamabad, Pakistan. It is a qualitative study based on constructivist grounded theory. Semi structured interviews of 16 participants, including five vascular surgical trainees, six vascular surgical consultants/supervisors/examiners, and five medical educationists were conducted, recorded and transcribed. Open and axial coding method was employed to identify recurring themes.

**Results:**

Six themes could be identified. (1) There was consensus among participants on deficiency in current assessment of surgical skills. (2) Most participants believed that this is a useful method, although four out of 16 participants believed that current methods were sufficient. (3) There was a unanimous opinion that its purpose should be initially formative assessment and later for summative assessment. (4) It was suggested that it is practical with logistic support; it can be made part of trainee’s record to be reviewed later; maybe by independent observers. (5) Participants believed that the logistic issue in term of equipment and trained manpower will be a challenge in implementing this mode of assessment. Other barriers included medicolegal and ethical issue and acceptability by the stake holders. (6) Participants also suggested remedies for the barriers.

**Conclusion:**

Video review of surgical procedures can improve assessment of operative skills of trainees provided it is used as formative tool initially with a need to overcome logistics, medicolegal and cultural barriers.

## INTRODUCTION

Training and exit exams of surgical specialties must be designed in such a way that they assess all domains of learning. Whereas it may be easy to assess the cognitive domains, it is difficult to adequately assess the psychomotor skills. At present, the College of Physicians and Surgeons Pakistan assesses surgical skills by a mix of direct and indirect methods: eLogbook and supervisor’s reports, DOPS (Directly Observed Procedural Skills), miniCX (Mini Clinical Exercise) and TOACS (Task Oriented Assessment of Clinical Skills). These methods leave much room for improvement.^1^-^3^ Therefore, we must look for some other tools to assess surgical skills of the trainees.

In recent years lot of research has been published on various ways to assess surgical skills. One of such methods is Video-based assessment of surgical skills, which if used in the right manner can provides a platform for objective, independent, and blinded assessment of surgical skills of not only the surgeons under training but also practicing surgeons.^4^-^6^ It will therefore overcome inherent limitations of assessments through indirect observation. Whether such a method will be acceptable in a surgical fellowship curriculum at CPSP or not, is unknown. In order to explore this topic, we conducted this study focusing on vascular surgery fellowship program in our own country.

**Fig.1:**
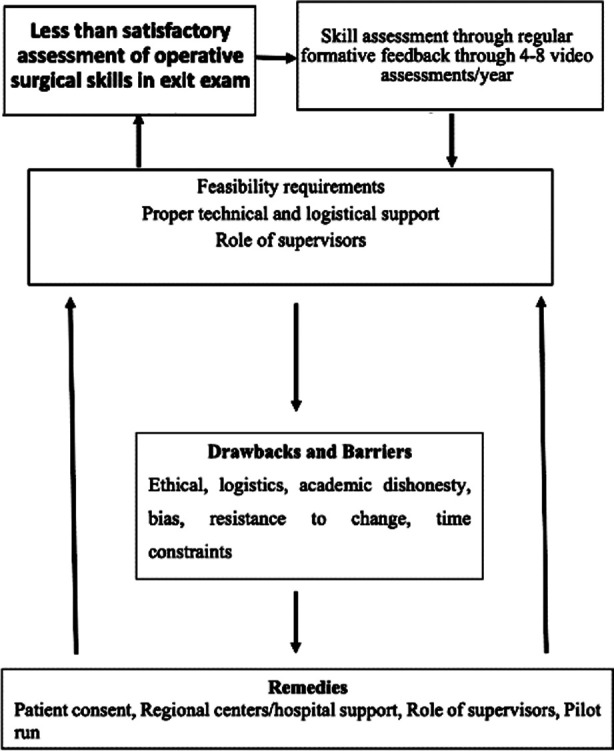
Conceptual framework of results showing how various identified themes connected.

This study aims to explore the feasibility of video assessment of technical surgical skills of FCPS vascular surgery trainees based on video recordings of the surgical procedures, with a view to introduce this in the curriculum.

## METHODS

This qualitative study was based on principles of Constructivist Grounded Theory and was carried out from 9^th^ April 2021 – 15^th^ July 2021 as a part of Masters in Health Professional Education thesis at Shifa Tameer e Millat University, Islamabad. Ethical approval from the Institutional Review Board was taken (Approval No 098-21dated 9^th^ April 21). The sample selection was purposive involving major stakeholders of the FCPS vascular surgery training program namely the FCPS trainees, the supervisors of FCPS and the CPSP/Medical educationists. After six interviews further theoretical sampling was done to purse clues that arose during analysis.

After a thorough literature search, a draft of the semi structured interviews was made and then vetted by two experts in the field. After taking informed consent, the interviews were conducted either face to face, on phone or online using Zoom® or Google Meet®. All the interviews were stored in the laptop in a secure location.

Interviews on predefined set of open-ended questions/domains were taken by author. They were recorded and field notes were taken. Iterative data collection through interviews and analysis with constant comparison was done.

Interview transcriptions were done manually as well as through software Amberscript® and Sonix®. These were checked and edited by the authors. Initially open coding was done to do an initial analysis to identify recurring themes and subthemes followed by axial coding. Trustworthiness and validity was achieved by following the recommendations by various authors^7^-^9^ to achieve credibility, dependability, transferability and confirmability.

## RESULTS

There were five vascular surgery trainees, seven vascular surgery consultants/supervisors/examiners and four medical educationists having affiliation with CPSP. Various themes and subthemes that emerged after analysis will be discussed one by one.

### Deficiency in Current Surgical Exit Exam:

All interviewees believed that the importance of assessing operative skills could not be overemphasized. The current exit exam method at CPSP was considered as good, but it lacks in the assessment of surgical skills of examinees. Interviewees shared their experience where they had often encountered qualified surgeons with poor technical surgical skills.

- ……*Not at all. It only assesses knowledge and very little assessment of skill…*.
- …… *Current methods focus very little on skills…*.

The participants described unsatisfactory role played by supervisors in giving timely feedback and fake entries made by trainees in haste were few of the reasons why they were dissatisfied with the exit exam and considered it deficient in assessing true technical surgical skills.

### Opinion About Assessment of Operative Skills Through Video Recording:

Most of the participants articulated in favor of assessment of surgical skills through video recording, they believed that video recording would have additional advantage over usual assessment methods as these could be recorded and reviewed at any convenient time. Both supervisors and trainees would be able to go back and see the recording step by step and identify mistakes. “*…… Can be played a number of times……”*.

Where most of the participants were in favor of video assessments, two were of the opinion that supervisors should play key role in training and carry out valid and regular workplace-based assessments in the form of DOPS. They believed that video assessment would only be an additional burden and just evidence for external reviewers of additional workplace-based assessment tool.

### Purpose of Assessment:

All study participants who were in favor of the video assessments strongly believed that since this assessment method has never been practiced before, therefore these should be used for improving skills via formative feedback only rather than summative assessment. Some believed that due to lack of adequate number of vascular surgery cases, standardization cannot be achieved. They agreed that this would be unfair to the trainee who has not been exposed and trained enough to record video which would have to be included in his/her summative assessment.

### Practicality and Method of Video Recording for Assessment:

Key informants of our study were of the opinion that the introduction of video assessments into the curriculum would require thorough groundwork and upfront planning which should be done by the regulatory body. For formal introduction into CPSP curriculum, they suggested uniformity in recording as well as assessment process, employing skilled manpower and a standardized procedure. All preferred a specified, neutral person to record to avoid bias and maintenance of secrecy in addition to supervisor.

Majority believed that a pilot study can be started as soon as possible in one or two recognized institutes. The number of recordings suggested for assessment in two years of training varied from four to eight at intervals of three to six months with main emphasis on basic and common index procedures.

### Drawbacks and Barriers in Video Recording Assessment Method:

Participants pointed to various challenges and hurdles in implementing this method. The foremost concern was lack of logistical support at CPSP centers/hospitals and non-availability of experts in handling hi-tec audiovisual aids. In addition, interviewees feared resistance from the CPSP, trainees and supervisors and use of unfair means for recording videos.

Majority of the participants were concerned about ethical issues related to recording, like patient confidentiality, informed consent and sharing of videos.

### Potential Remedies for Barriers:

Participants suggested few methods to overcome barriers particularly ethical and logistics issues. They were of the opinion that the use of written informed consent of all stakeholders would be solution to overcome any medico legal and ethical problems. They suggested that CPSP regional centers or hospitals should fund this facility and recordings maybe carried out at those stations or hospitals under supervision of supervisors initially and later by neutral experts to avoid chance of video manipulation too. A pilot study should first be carried out to sensitize trainees and supervisors before incorporating in the curriculum.

## DISCUSSION

Video recording can be used to assess surgical skills and to give feed back to the trainees. This method is currently used in different forms in many countries. Feasibility of its introduction as a part of a surgical fellowship program has not been studied so far. The current studied tried to bridge this gap.

In our study, various themes could be identified after going through all the 16 interviews. The first theme on which there was consensus was inadequacy of current system of assessment of surgical skills across the FCPS training. Surgical logbook or lately eLogbook is the main tool currently being used in the FCPS training to assess the surgical skills. It is an indirect method for surgical skill assessment which only tells us how many procedures the trainee has supposedly performed or was part of the procedures. It doesn’t tell us how well the trainee has performed the procedures and is he or she fully competent to perform them independently.^10^,^11^ Moreover, some participant admitted that many a times logbook entries were written towards the end of the training therefore the correctness of these entries can be debated. As Memon et al suggested^1^ that this is “just a list of procedures performed or assisted by the trainees”. In one study by Iqbal et al.,^12^ it was found that the trainee have high level of satisfaction with the new system of eLogbook. Although in this mixed method study the main complaint of the trainees was lack of timely feedback by the supervisors. In another study by Gondal et al.^13^ CPSP supervisors were satisfied by the eLogbook as tool to improve and monitor training. But this was a simple cross-sectional study and did not investigate the problems and barriers in effective use of the eLogbook. In a qualitative study by Ullah et al.^2^, the perceptions of supervisors using the eLogbook was assessed. Although most of the supervisors agreed with the importance and utility of eLogbook, they also admitted that they do not use it regularly, use it to just ‘approve the entries’, or either do not give the feedback or give it when requested by the trainee to do so. One of the supervisors said that sometimes the supervisor shares the password with the trainee to approve the entries. Clearly the onus of improvement in this system lies with the supervisor. Because of lack of time and lack of insight into the importance of the system many supervisors have a casual approach towards this tool of assessment and as a result its utility is questionable.

Recently CPSP has introduced two WPBA tools. They are Mini CEX and DOPS. The educational value of these tools is well established *if used properly*. It is said that you need 15 minutes to do the exercise and five minutes for the feedback. But in fact, the exercise requires much more time. These WPBAs are perceived as an additional workload and therefore this attitude diminishes their educational impact.^14^ This fact is reflected in the study by Ullah et al.^2^ and also in interviews in our study. Interestingly in a study by Shalhoub and colleagues^15^, the UK training system is facing the same problem with supervisors not giving timely feedback, or taking it casually. Some of the trainees admitted that they fill the Procedure Based Assessments (PBA’s) themselves. Moreover, there is a potential conflict of interest if the feedback is only given by the educational supervisor.^15^ As one of the authors observed that the biggest challenge to implementation of PBAs is change in culture, and a sense of responsibility of supervisors to rise up to these expectations.^16^ It is probably because of this reason that the ISCP (The Intercollegiate Surgical Curriculum Programme (ISCP)) in the UK has removed the compulsory requirement of fixed number of PBAs during training.^17^

Opinion of participants about using video recordings to assess surgical skills was mixed. Four considered live feedback on WPBA better than the video recorded ones. Rest of the participant had a more favorable view of this mode of assessment and considered it doable. One of the participants pointed out that the videos are there for reviewing as many times as one needs. This advantage is also mentioned in one of the review article by Goldenberg.^18^

All participants were of the view that video-based assessment should be for formative purposes and were against its use for summative assessment due to lack of validity and reliability. This has been mentioned by many authors who advocate using video recording for feedback and not to make a pass/fail decision.^19^,^20^ A summative assessment needs to have more validity than that used for formative assessment.^21^ It is because of these difficulties that there is no reliable study assessing the validity of surgical skill assessment based on video recording for summative purposes.^20^,^22^ But with advances in technology of video cameras, the storage ability of the equipment, the ease of transfer over Internet, introduction of crowd sourcing and Artificial Intelligence to assess the technical aspects of video recording, it is clear that in future video recordings-based assessment will be common and will be used for various purposes both formative and summative.^23^,^24^

The medicolegal aspect of video recording is something that threatens the surgical team. Surgeons vary in their performance. There can be more than one way to do a procedure, this does not mean that one way is better than the other. In order to avoid unnecessary litigation we need to set standards for every procedure.^25^ Moreover, the mere awareness that the videos are being recorded and could be used in case of litigation as medical record, can have negative influence on the surgical team.^25^ Some authors believe that although it appears that the videos will be used against surgeons, they can actually save a surgeon, if he or she can demonstrate that all the necessary steps were taken during the operation.^26^ Most of the participants were not clear about potential medicolegal usage in court of law.

Time constraint for both trainee and supervisors is one of the major barrier to implement any form of feedback. The same concern is expressed by other authors as well.^18^,^24^ Technology has come to our help in this case too. The availability of crowd sourcing for reviewing videos and use of AI can potentially solve the problem.^20^ One paper found that video assessment done by crowd sourcing (like Amazon Mechanical Turk) can give the feedback within four hours as compared to up to two weeks by supervisors^27^ although it may not be as good as the experts.

### Limitations:

Inclusion of technical and medicolegal experts and personnel from hospital administration would have been useful. Since so much emphasis is laid on the role of supervisors, it remains to be explored that why do supervisors do not fulfil their responsibilities as expected and what is their perspective. Moreover, a pilot study at one or two institutes would have shed light on practicability of the current proposal. This however would need a separate study.

## CONCLUSION

Current methods of assessment of operative skills in the FCPS training program of the CPSP need improvement. It is possible to assess trainees’ surgical skills based on the video recording of an operation performed. This will require a significant logistic arrangement. This assessment should start as formative assessment with a pilot study at one or two institutes. Introduction of such a component in the FCPS curriculum is a challenge. However, with the help of all the stakeholders, that is, trainees, supervisors and CPSP it is possible to gradually introduce it in the curriculum.

### Authors` Contributions:

**AMB:** Principal Investigator, Conception of design, collection and analysis of data, drafting of article, responsible for integrity and correctness of research.

**SA:** Conception of design, participation in collection and analysis of data (transcription of interviews, coding and analysis of transcribed interviews), drafting of final manuscript, proof reading.

**SM:** Design conception, critical review and approval of final work.
